# Transcriptomic and life history responses of the mayfly *Neocloeon triangulifer* to chronic diel thermal challenge

**DOI:** 10.1038/s41598-020-75064-y

**Published:** 2020-11-05

**Authors:** Hsuan Chou, Dereje D. Jima, David H. Funk, John K. Jackson, Bernard W. Sweeney, David B. Buchwalter

**Affiliations:** 1 Graduate Toxicology Program, Department of Biological Sciences, North Carolina State University, 850 Main Campus Drive, Rm1112, Raleigh, NC 27695 USA; 2 Center of Human Health and the Environment, North Carolina State University, Raleigh, NC 27695 USA; 3 Stroud Water Research Center, Avondale, PA 19311 USA; 4 Bioinformatics Research Center, North Carolina State University, Raleigh, NC 27695 USA

**Keywords:** Genetics, Physiology, Ecology, Environmental sciences

## Abstract

To better understand the effects of transient thermal stress in an aquatic insect, we first identified static temperatures associated with fitness deficits, and then reared larvae from egg hatch to adulthood under diurnally variable regimens including daily forays into deleterious temperatures. We sampled mature larvae at the coolest and warmest portions of their respective regimens for RNA-seq analysis. Few transcripts (28) were differentially expressed when larvae oscillated between favorable temperatures, while 614 transcripts were differentially expressed when experiencing daily transient thermal stress. Transcripts associated with *N*-glycan processing were downregulated while those associated with lipid catabolism and chitin turnover were significantly upregulated in heat stressed larvae. An across-regimen comparison of differentially expressed transcripts among organisms sampled at comparable temperatures demonstrated that the effects of daily thermal stress persisted even when larvae were sampled at a more optimal temperature (806 differentially expressed transcripts). The chronically stressed population had reduced expression of transcripts related to ATP synthesis, mitochondrial electron chain functions, gluconeogenesis and glycolytic processes while transcripts associated with cell adhesion, synaptic vesicle transport, regulation of membrane potential and lipid biosynthesis increased. Comparisons of constant vs. variable temperatures revealed that the negative consequences of time spent at stressful temperatures were not offset by more time spent at optimal temperatures.

## Introduction

Temperature is among the most important factors that determine the distribution and performance of ectothermic species^1–8^. In most aquatic ecosystems, organisms experience thermal regimes that include distinct diel cycles that are imbedded within the more commonly studied seasonal cycle^9–12^. As the global climate changes and human activities alter the thermal regimes of freshwater ecosystems^13–16^, it is increasingly likely that organisms are subjected to stressful temperatures on different temporal scales^17^(e.g. hourly, daily, seasonally, annually) that differentially affect physiological processes, developmental trajectories, life history outcomes, and ultimately species distributions^18^.

Insects play critical ecological roles and are the most widely used faunal group to evaluate ecological conditions of freshwater environments^19–21^. While insect thermal biology has been broadly studied, less has focused on aquatic insects. At the seasonal/annual scale, it appears that aquatic insects typically adhere to the temperature size rule (TSR)^3,5,22,23^, though some exceptions have been noted^22^. For a given species, relatively warm temperature accelerates growth and developmental rates, with development time decreasing at a disproportionately faster rate than biomass accumulation. As a result, warmer conditions produce smaller, less fecund individuals, relative to cooler temperatures, which produce larger, more fecund individuals. In life cycle rearing studies at constant temperatures with the mayfly *Cloeon dipterum*, Sweeney et al.^8^ defined a thermal “acclimation zone” where development and growth rates changed consistently with increasing temperature while degree-day requirements to complete metamorphosis were constant. Rearing at temperatures warmer than the thermal acclimation zone significantly reduced survival and fitness.

As thermal regimes continue to change, it is increasingly likely that some species will spend greater portions of their life cycle at daily high temperatures that are outside of the thermal acclimation zone. While some research in the thermal biology field has begun to incorporate transient thermal stress in recent years^24^, the fitness consequences of exposure to diel temperature fluctuations in insects still remains poorly understood. Understanding the physiological processes that occur under repeated, transient thermal stress are especially important because this situation is likely common as conditions warm.

Recent efforts have increased our knowledge of the thermal biology of mayflies and the development of a lab-reared mayfly model (*Neocloeon triangulifer* (Ephemeroptera: Baetidae) for physiological and ecological studies^25–35^. We have developed a better understanding of both short-term^36,37^ and long-term^38^ thermal challenge in *N. triangulifer,* but the more ecologically relevant situation of long-term development in a diel thermal regime with daily excursions out of the acclimation zone and into stressful temperatures has remained unstudied.

In this paper, we examine the consequences of daily, transient exposures to thermally challenging temperatures. We first used constant temperature rearing experiments (from egg hatch to adulthood) to identify a physiologically stressful and fitness-reducing temperature – in this case 28 °C which clearly fell outside the thermal acclimation zone for *N. triangulifer*. We then established variable temperature regimens that included a brief (hours), daily exposures to 28.5 °C. We used an RNA-seq approach to compare gene expression profiles of mature larvae sampled at the low and warm temperatures of their respective daily thermal regimes to better understand how short-term forays into sub-optimal (warm) conditions affected gene expression. We also made comparisons of gene expression profiles across thermal regimens to better understand how signatures of transient but chronic thermal challenge are retained at the transcriptomic level, even at “recovery” temperatures. We link these results to life history outcomes and highlight the important role of molting in mediating the thermal performance.

## Materials and methods

### Mayfly rearing

The parthenogenetic mayfly *N. triangulifer* (WCC-2 clone isolated from White Clay Creek, (Patent US5665555)) were reared at the Stroud Water Research Center (SWRC; Avondale, PA). Newly hatched eggs of *N. triangulifer* were partitioned into rearing jars using natural stream water from White Clay Creek (WCC) and natural WCC periphyton as a food source as described elsewhere^39^. Mayflies were reared at four constant temperature treatments (22°, 26°, 28°, and 30 °C ± 0.1 °C) and three variable temperature treatments with a 5 °C daily oscillation (0.5 °C/h) from 19.5 °C to 24.5 °C, mean = 22 °C (Regimen 1- diel fluctuation within the thermal acclimation zone) 23.5 °C to 28.5 °C, mean = 26 °C (Regimen2-diel fluctuation outside the thermal acclimation zone), and 25.5–30.5 °C, mean = 28 °C (Regimen 3 -diel fluctuation further outside the thermal acclimation zone). In each rearing condition, 5–11 replicate jars were used, and each jar contained 50 larvae. Temperature starts to rise at 6:30 am and reaches high soak at 4:30 pm for 2 h. Temperature starts to fall at 6:30 pm and reaches low soak at 4:30 am for 2 h. All temperature treatments were exposed 15:9 (L:D) photoperiod that began at 5:00 am and ended at 8:00 pm, simulating a light regime near the summer solstice at ~ 40°N). Survivorship, development time, and adult body size (fitness) were recorded for each replicate jar. Survivorship was the percentage of 1st-instar larvae surviving to the adult stage. These data were arcsine transformed for statistical analysis. Development time was the number of days from the start of the experiment (egg hatch) to the median day of adult emergence from a given replicate jar. Adult body size was mean individual biomass of emergent adults from a given replicate jar, dried at 60 °C for 48 h.

### Larval rearing for molt counting

Hatchlings (< 12 h old) of *N. triangulifer* (WCC-2 clone) were reared from first instar larvae to the adult (subimago) stage in incubators (Thermo Scientific, MA) held at 18 °C and 26 °C ± 0.05 °C (trial 1), 22 °C and 26 °C ± 0.05 °C (trial 2) constant temperatures at NCSU. To conveniently monitor and collect mayfly exuviae, a single larva was put in a well containing artificial soft water (ASW) in a 12 well plate (Genesee Scientific, CA). Each temperature treatment contained three replicates (12-well plates). Larvae were transferred into larger 6-well plates as they developed. Simulated daylight was provided by fluorescent “grow lights” and all experiments involved a 15:9 h light:dark cycle. Food was provided every other day using periphyton shipped from SWRC. Water was filled and kept at the surface of each well to ensure larvae were oxygenated. Larvae molt count was conducted every day and exuviae were removed from wells.

### Mayfly larval sampling for RNA-seq

Because temperature strongly influenced development time, it was essential for larvae in the different thermal treatments to be sampled at comparable developmental stages. While there are no perfect anatomical features that would allow for this developmental synchronization to be precise, the presence of developed but not darkened wing pads was used as a visual guide to sampling at the low soak and high soak temperatures of the respective thermal regimes. For the Regimen 1 group larvae were sampled on day 21.7 and 22.2 of larval development. The median larval emergence time for this thermal treatment was 23.7 days. Thus, we estimate that larvae from the 22 °C group were sampled at 92–94% completion of larval development. For the Regimen 2 group, larvae were sampled on day 18.2 and 18.7 of larval development. The median emergence time for this thermal treatment was 20.1 days. Thus, we estimate that larvae from the Regimen 2 group were sampled at 91–93% completion of larval development. Upon collection, all samples were flash frozen in liquid nitrogen and stored at − 80 °C freezer. All samples were packaged with dry ice and sent with overnight shipping to North Carolina State University (NCSU; Raleigh, NC) for RNA-seq at the NCSU Genomic Sciences Laboratory (GSL). Biological replicates were n = 4 for RNA-seq and n = 6 qPCR analysis.

### RNA-seq and data analysis

To prepare samples for RNA-seq studies, total RNA was isolated from *N. triangulifer* following the SV Total RNA Isolation System protocol (Promega, WI). RNA quality was assured with Bioanalyzer RNA nano chips (Agilent, CA); Truseq libraries (Illumina, CA) were prepared; and 16 libraries were sequenced with paired-end reads using the Illumina NextSeq 500 at the NCSU GSL.

### Transcriptome assembly

Transcriptome assembly and differential expression were performed in consultation with Bioinformatics Core at NCSU Center for Human Health and the Environment (CHHE). The quality of sequenced data was assessed using FastQC application, and the adapter sequence and quality trimming was preformed using Trimmomatic version 0.36^40^ with the following parameters (HEADCROP:12, LEADING:20, TRAILING:20, SLIDINGWINDOW:30:30, MINLEN:40). Transcriptome de novo assembly were conducted using trinity^41^. The transcriptome sequences were annotated using BLAST + command line utility (blastx and blastp; E-value cutoff 1e^-5^ and max target sequence -1)^42^ and the Trinotate pipeline (https://trinotate.github.io/). Since there was no *N. triangulifer* reference genome and mapping reads to another mayfly, *E. danica* resulted with less than 1% unique mapping, the raw reads were mapped back to the de novo assembled transcriptome. The count matrix was generated using align and estimate abundance Perl script in the Trinity software package using RSEM abundance estimation method and bowtie2 aligner.

### Pairwise analysis

Differentially expressed genes were determined using the R package DESeq2^43^. The Count data were normalized for sequencing depth and RNA composition, specifically the counts divided by sample-specific size factors determined by median ratio of gene counts relative to geometric mean per gene.

We fitted a linear model using the treatment levels, and differential expressed genes were identified after applying multiple testing correction using Benjamini–Hochberg procedure^44^ , padj < 0.05. Significant differentially-expressed genes (UniprotKB IDs) were assigned for Gene Ontologty (GO) analysis through comparison with annotated protein sequences from *Drosophila melanogaster* with online web tool PANTHER (https://geneontology.org/). Expression results were validated by conducting qPCR on a subset of genes (Fig. 4). Pathway analyses were conducted with two online database: reactome pathway analysis via PANTHER and KEGG pathway analysis via DAVID (https://david.ncifcrf.gov/summary.jsp). Both analyses showed similar result, and data presented in this study are shown as reactome pathways via PANTHER.

### qPCR validation

To validate the expression results from RNA-seq analysis, we selected 11 genes for confirmation by conducting qPCR. We selected genes randomly from several functional categories of interest, including a mix of up- and down-regulated genes by our treatments. All primers were designed with IDT PrimerQuest Tool (https://www.idtdna.com/Primerquest/Home/Index) with the following parameters: length of 18–22 nt, melting temperature of 60 °C and product size of 150–220 bp. Primers were synthesized by Life technologies, USA and were tested using conventional PCR and gel electrophoresis for correct size product. cDNA for qPCR was generated from aliquots of the same RNA samples used for RNA-seq with 2 additional biological replicates for each treatment. First strand cDNA was synthesized from the same amount of each total RNA by MultiScribe MuLV reverse transcriptase using random primers (Applied Biosystems, (ABI), CA) and all thermocycling was done using a Bio-Rad iCycler (Bio-Rad, CA). The resulting cDNA samples were diluted 4 × before analysis and stored at − 20 °C. Quantitative real-time PCR (qRT-PCR) was performed on an ABI Prism 7700 Sequence Detection System (Applied Biosystems (ABI), CA) using default parameters. Amplification mixtures consisted of 5 µL of SsoAdvanced Universal SYBR Green Supermix (Bio-Rad, CA), 10 µM primers, 20 ng template cDNA and nuclease free water in a total volume of 10 µL. qRT- PCR conditions were 2 min at 94 °C, followed by 40 cycles at 95 °C for 30 s, 60 °C for 30 s, and 72 °C for 30 s. Relative expression of each amplicon was calculated by the corrected delta delta Ct method (Pfaffl 2001), with *EF1α* serving as a reference gene^38^. Relative levels of *EF1α* were confirmed to be approximately equal across all treatments.

## Results

### Life history responses in constant temperature regimes.

**Figure 1:**
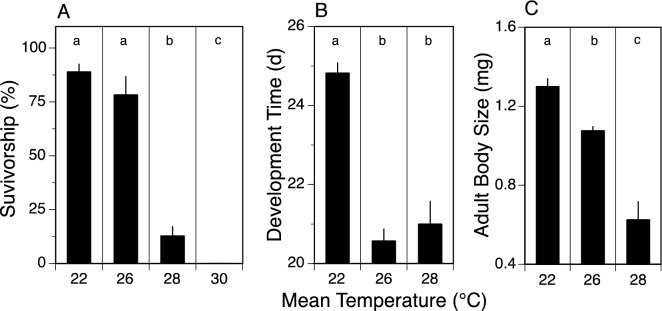
Measures (mean ± 1SE) for survivorship (**A**), development time (**B**), and adult body size (dry weight) (**C**) across four constant temperature treatments (22, 26, 28, and 30 °C), with statistically significant differences (1-way ANOVA with Tukey’s multiple comparison test, *p* ≤ 0.05) between treatments indicated by different letters over bars (**a**,**b**,**c**). Survival, development time, and adult body size identify 28 °C as detrimental relative to 26 °C and/or 22 °C. Each mean represents the results for 7–9 replicate rearing jars, each containing 50 larvae.

In constant temperature treatments, survivorship was 89% at 22° and 78% at 26 °C, but decreased to only 12.8% at 28 °C. No larvae survived to adulthood at 30 °C (Fig. 1A). Development time decreased from 24.8 d at 22 °C to 20.6 d at 26 °C, and then was unchanged (20.6 vs 21.0 d) between 26 and 28 °C (Fig. 1B). Adult body mass decreased gradually from 1.3 mg at 22 °C to 1.1 mg at 26 °C and 0.6 mg at 28 °C (Fig. 1C). Note that adult body size (as dry mass) is an excellent predictor of fecundity^25^; Funk, Jackson, Sweeney, unpublished data), and therefore individual fitness. When comparing 26° and 28 °C, the dramatic increase in mortality, absence of a decrease in development time, and decrease in adult body size establishes 28 °C as a clearly detrimental temperature based on these life history outcomes.

**Figure 2:**
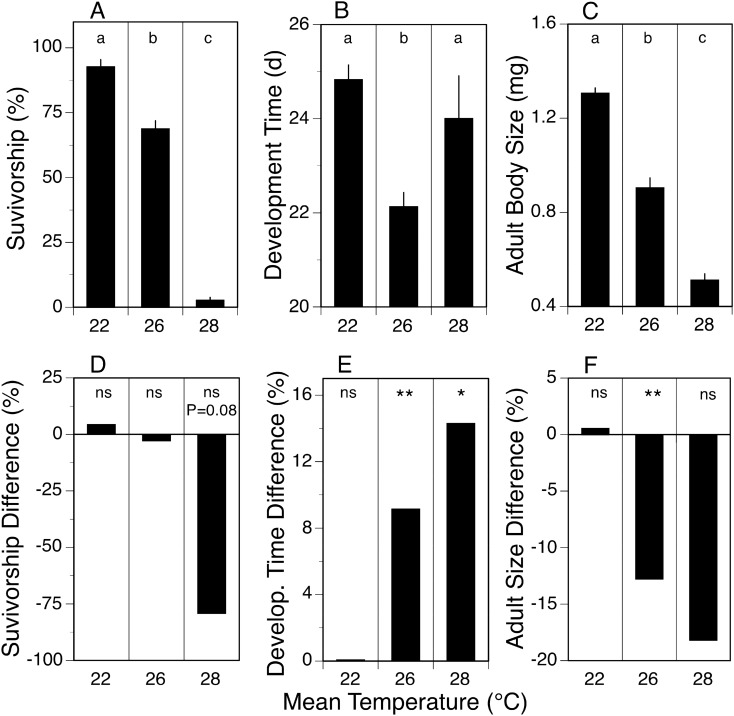
Measures (mean ± 1SE) for survivorship (**A**), development time (**B**), and adult body size (dry weight) (**C**) across three variable temperature treatments (22, 26, and 28 °C). Each variable temperature treatment consisted of means from 4 replicate rearing jars, each containing 50 larvae. Statistically significant differences (1-way ANOVA with Tukey’s multiple comparison test, *p* ≤ 0.05) between variable treatments indicated by different letters over bars (**a**,**b**,**c**). Difference between constant and variable temperature treatments expressed as % difference (**A**–**C** relative to Fig. 1A–C) for survivorship (**D**), development time (**E**), and adult body size (**F**). Statistical significance (Student’s t-test) indicated by ns (*p* > 0.05), * (*p* ≤ 0.05) or ** (*p* ≤ 0.01).

We used this information to rear *N. triangulifer* under three variable temperature regimens (Regimen 1: diel minimum of 19.5 °C and maximum of 24.5 °C, mean 22 °C; Regimen 2: 23.5–28.5 °C, mean 26 °C; Regimen 3: 25.5–30.5 °C, mean 28 °C) from egg hatch to adult. The responses for survivorship, development time, and body size in variable temperature treatments were similar to what was observed in constant temperature treatments – survivorship, development time, and body size decreased as mean temperature increased (Fig. 2A–C).

### Molting experiment

We reared mayflies in well plates so that we could monitor individual larvae on a daily basis and quantify the total number and frequency of molts. In two separate trials (trial 1 comparing 18 °C and 26 °C, and trial 2 comparing 22 °C and 26 °C), we found that there was no statistical difference in the total number of molts required to complete larval development within each trial. In trial 1, larvae averaged 13.4 ± 1.9 and 14.3 ± 1.3 molts at 18 °C and 26 °C, respectively. In trial 2, larvae averaged 13.5 ± 1.4 and 11.8 ± 1.6 molts respectively. Thus, because warmer temperature reduces development time and body size, there is less time between molts (e.g. 13 molts in 30 d vs 21 d), and molts later in development occur at a smaller size.

### Life history outcomes in variable vs constant temperatures

The response to being exposed to a variable temperature regime of ± 2.5 °C versus a constant temperature regime depended on the mean temperature examined, and the life history parameter being measured. The variable temperature regime had no significant effect on survivorship, development time, and adult body size when mean temperature was 22 °C (Fig. 2D,E,F). In contrast when mean temperature was 26 °C, the variable temperature regime had no significant effect on survivorship (Fig. 2D), but development time was 9.1% longer (Fig. 2E) while adult body size was 12.8% less (Fig. 2F) than in the constant temperature treatment. Finally, survivorship in the variable temperature treatment at 28 °C was 79.2% lower (nearly significant at *p* = 0.08), development time was 14.3% greater, and adult body size was 18.2% smaller relative to the constant temperature treatment at 28 °C. The differences between the constant and variable temperature treatments at 28 °C was not always significant due to low survivorship in both treatments (i.e., few individuals survived). These results show that daily exposure of larvae to brief periods of 24.5 °C had no impact on the life history traits measured, but brief periods of 28.5 °C in the variable 26 °C treatment had a negative impact on development time and adult body size, and brief periods of 30.5 °C had a negative impact on survivorship, development time, and adult body size. Thus, larvae in warmer, variable temperature treatments responded negatively (e.g., reduced survivorship and adult size, increased development time) relative to constant temperature treatments, and the negative response intensified as temperature increased. The differences between variable and constant temperature treatments at 26 °C highlight that time spent at 23.5 °C does not compensate for the negative impact of a brief exposure to 28.5 °C.

### Differential gene expression in variable thermal regimes

**Figure 3:**
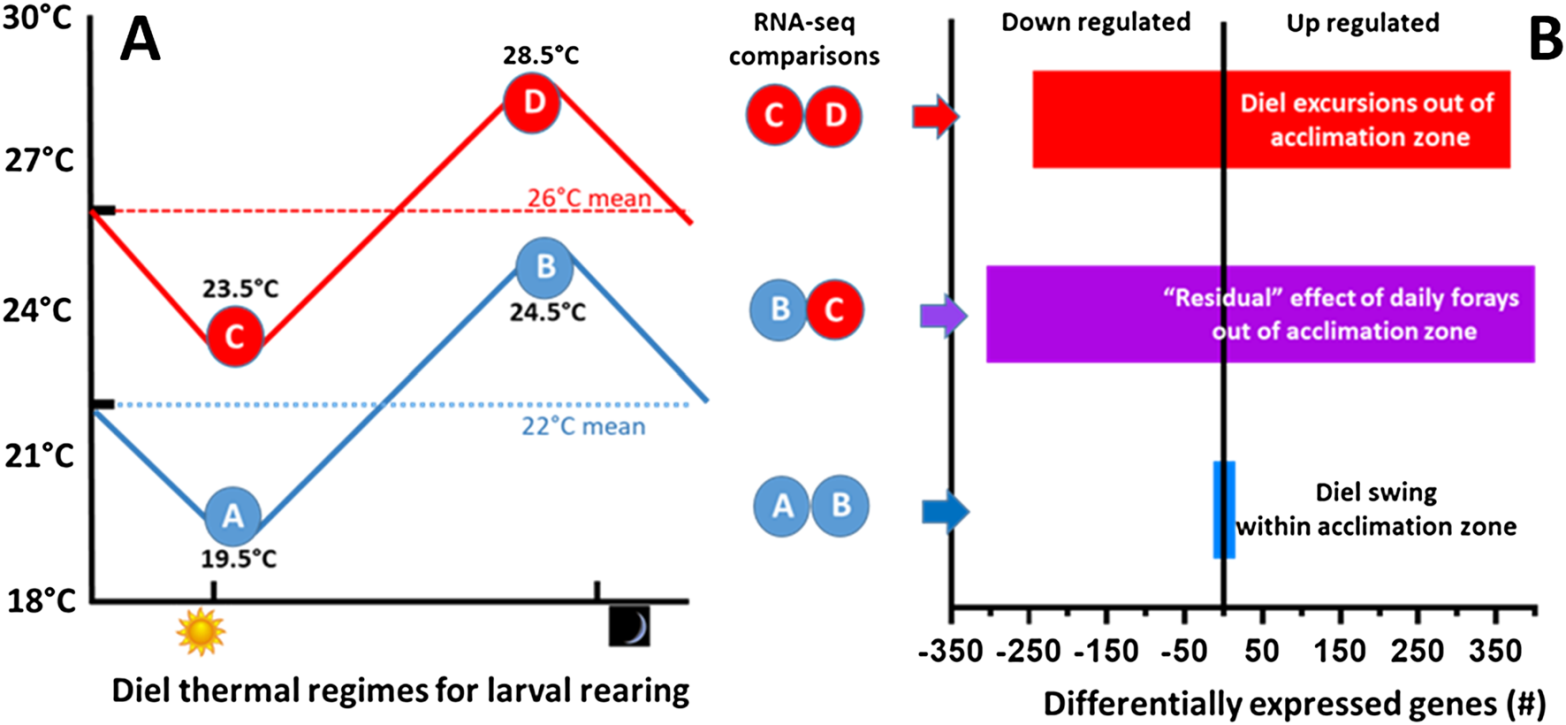
(**A**) Thermal regimens for rearing and sampling strategy for RNA-seq analyses. Circled letters represent the sampling temperatures for mature larvae reared for their entire larval development period. The 22 °C mean temperature regime (regimen 1) oscillated daily between 19.5 and 24.5 °C. The 26 °C mean temperature regime (regimen 2) oscillated daily between 23.5 and 28.5 °C. (**B**) The numbers of differentially expressed genes associated with each pairwise comparison.

To better understand the influence of variable (diel) thermal regimes on global RNA expression patterns (Fig. 3), we first compared RNA-seq data in larvae that were reared entirely within the thermal acclimation zone (Regimen 1: diel cycles between 19.5 °C and 24.5 °C, daily mean 22 °C) (See Table S1). Remarkably few transcripts (28) were differentially expressed in larvae sampled at 19.5 °C vs. 24.5 °C that met our criteria of a false discovery rate (FDR) < 0.05. Of the 28 differentially expressed transcripts, 7 were upregulated and 19 were downregulated, but only 17 were named or have known or inferred function (A vs B in Fig. 3B). These few genes with known function (Table S1) are largely associated with functions related to circadian clock and/or visual functions such as rhodopsin-specific isozyme, peptidyl-prolyl cis–trans isomerase (protein folding, possible roles in co-chaperone activities and steroid hormone receptor trafficking), However, no thermal stress related responses were noted in this comparison.

**Figure 4:**
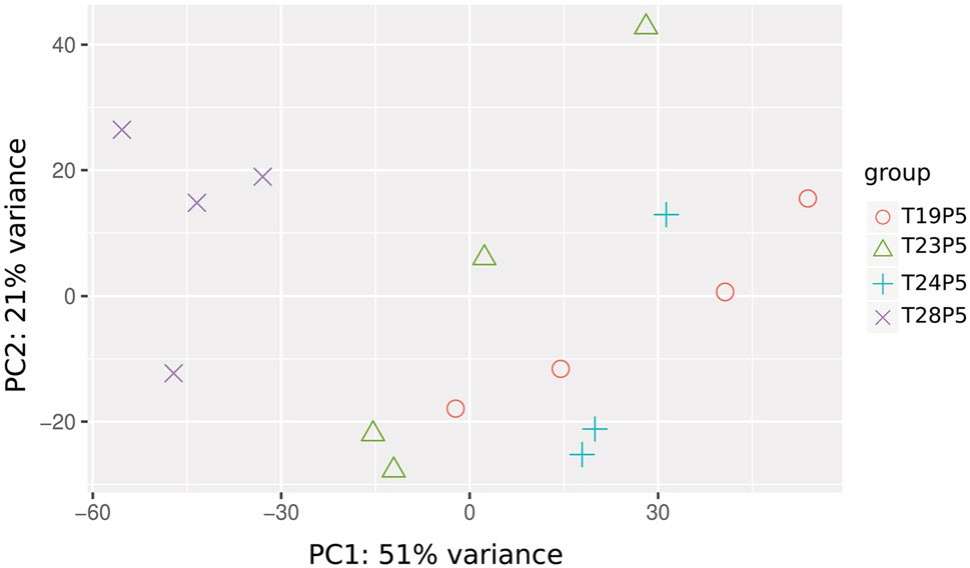
PCA plot showing similarity within Regimen 1 (19.5 and 24.5 °C) whereas the Regimen 2 shows strong effects of exposure to 28.5 °C. This transient 28.5 °C exposure appears to influence gene expression in the 23.5 °C group.

In contrast, when larvae were subjected to daily excursions out the thermal acclimation zone (Regimen 2: diel fluctuations between 23.5 and 28.5 °C, daily mean 26 °C), we identified 514 differentially expressed genes, with 369 (60%) upregulated and 245 (40%) downregulated genes (C vs D in Fig. 3B) (Table S2). A cross regimen (regimen 3) comparison was made between larvae sampled at 24.5 °C in regimen 1, and larvae sampled at 23.5 °C in regimen 2. Here we identified 806 differentially expressed genes, with 501 (62%) upregulated and 305 (38%) downregulated genes (B vs C in Fig. 3B) (Table S3). A principal components analysis of the gene expression data (Fig. 4) shows similarities within regimen 1. However, within regimen 2, responses to 28.5 °C are clearly separate from 23.5 °C. For completeness, the other pairwise comparisons are provide in Tables S4−S6.

### Functional comparisons of diel fluctuations outside of the thermal acclimation zone

**Figure 5:**
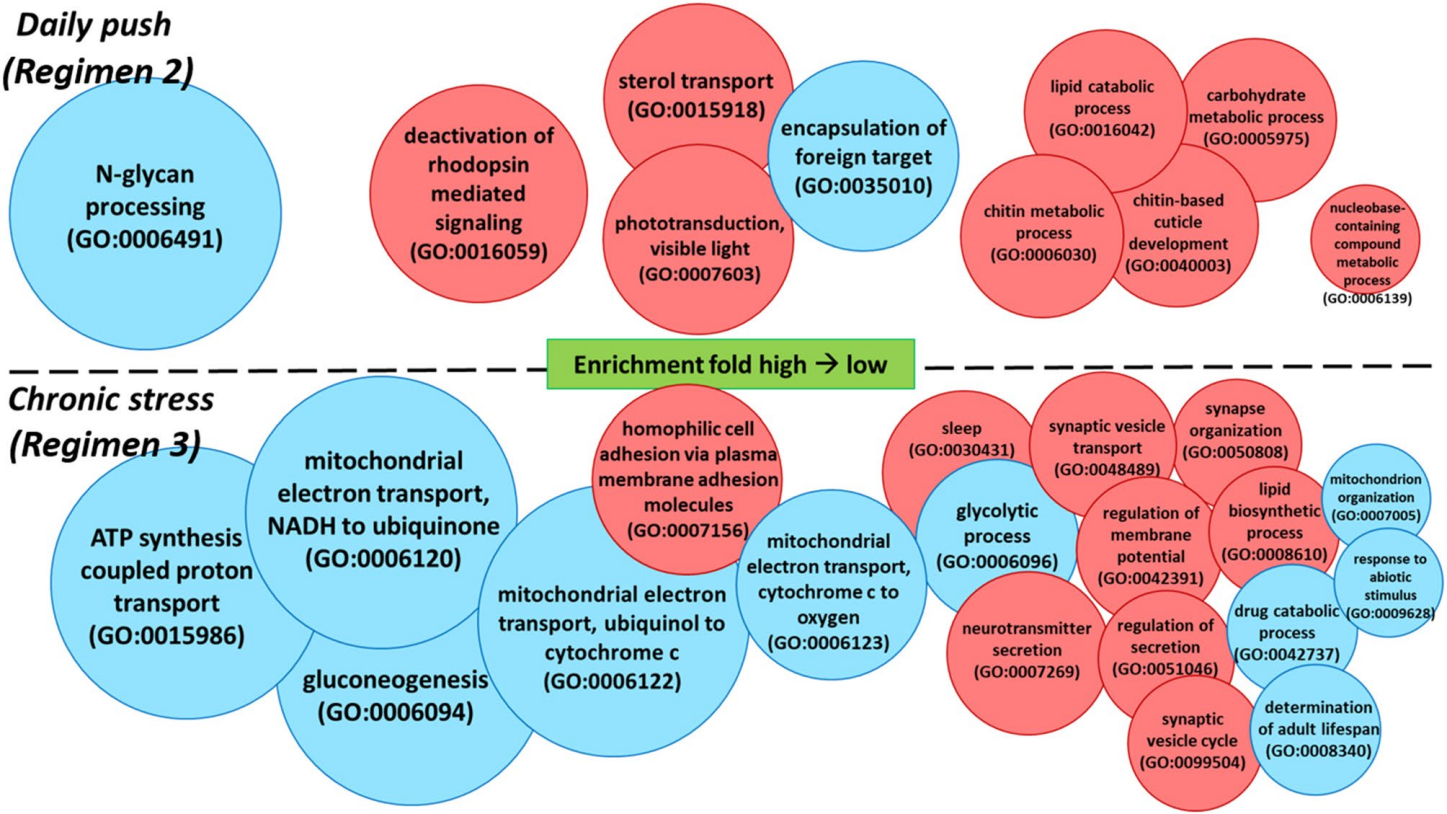
Gene ontology (GO) enrichment analysis results showing GO enrichment analyses for biological processes affected in regimen 2 (top panel and C–D comparison from Fig. 3), and regimen 3 (bottom panel, B–C comparison from Fig. 3). Biological functions are represented as upregulated (red) and downregulated (blue) and are size scaled based on calculated fold enrichment of our differentially expressed genes dataset compared to drosophila database.

To further analyze and distinguish between functional groups of the differentially expressed genes that responded to transient daily thermal challenge (Fig. 3; Regimen 2), we separated the gene ontology (GO) enrichment analysis into lists of up- and down-regulated genes (Table S3). Comparing the daily high soak (28.5 °C) to low soak (23.5 °C) samples in regimen 2, GO enrichment analysis through web-based tool (PANTHER) identified up-regulated genes associated with GO enrichment analyses for biological processesof sterol transport, chitin metabolic process, lipid catabolic process, chitin-based cuticle development, carbohydrate metabolic process and nucleobase-containing compound metabolic process (Fig. 5 upper panel, red circles). Other up-regulated processes were associated with clock/time of day as described above and included deactivation of rhodopsin mediated signaling and phototransduction. The GO enrichment analysis identified down-regulated genes with major GO enrichment analyses for biological processesin *N*-glycan processing (packaging of monosaccharides) and encapsulation of foreign target (Fig. 5 upper panel, blue circles). Reactome pathway analysis combining both up- and down-regulated genes suggested that pathways involved in lipid and lipoprotein metabolism are significantly enriched during thermal challenge. Of the genes residing in the pathway, 63% were up-regulated while 37% were down regulated.

### Functional comparisons of the residual effects of chronic but transient thermal stress

To assess the lingering or persistent effects of transient daily forays into challenging temperatures, we compared RNA-seq profiles of larvae sampled at 23.5 °C (the low soak (cool) portion of regimen 2) with those sampled at 24.5 °C (the high soak (hot) portion of regimen 1) (See B-C comparison from Fig. 3) (Table S4). GO enrichment analysis identified up-regulated genes associated with several biological process such as neurotransmitter secretion, synaptic vesicle transport/cycle/organization, and lipid biosynthetic process (Fig. 4, lower panel, red circles). Down-regulated genes were enriched in GO enrichment analyses for biological processes such as ATP synthesis coupled proton transport, mitochondrial electron transport gluconeogenesis, glycolytic process, drug catabolic process, and determination of adult lifespan (Fig. 4, lower panel, blue circles). Reactome pathway analysis combing both up and down-regulated genes suggested enrichment in pathways including acetylcholine neurotransmitter release cycle, formation of ATP by chemiosmotic coupling, complex l biogenesis, PLC beta mediated events and glycolysis. Of the pathways involved, 80% and 71% of the genes are up-regulated in the acetylcholine neurotransmitter release cycle and the PLC beta mediated events, respectively. Genes involved in metabolism pathways- formation of ATP by chemiosmotic coupling, complex l biogenesis and glycolysis, are all strongly down-regulated (92%, 84% and 85%, respectively).

### qPCR confirmation

**Figure 6:**
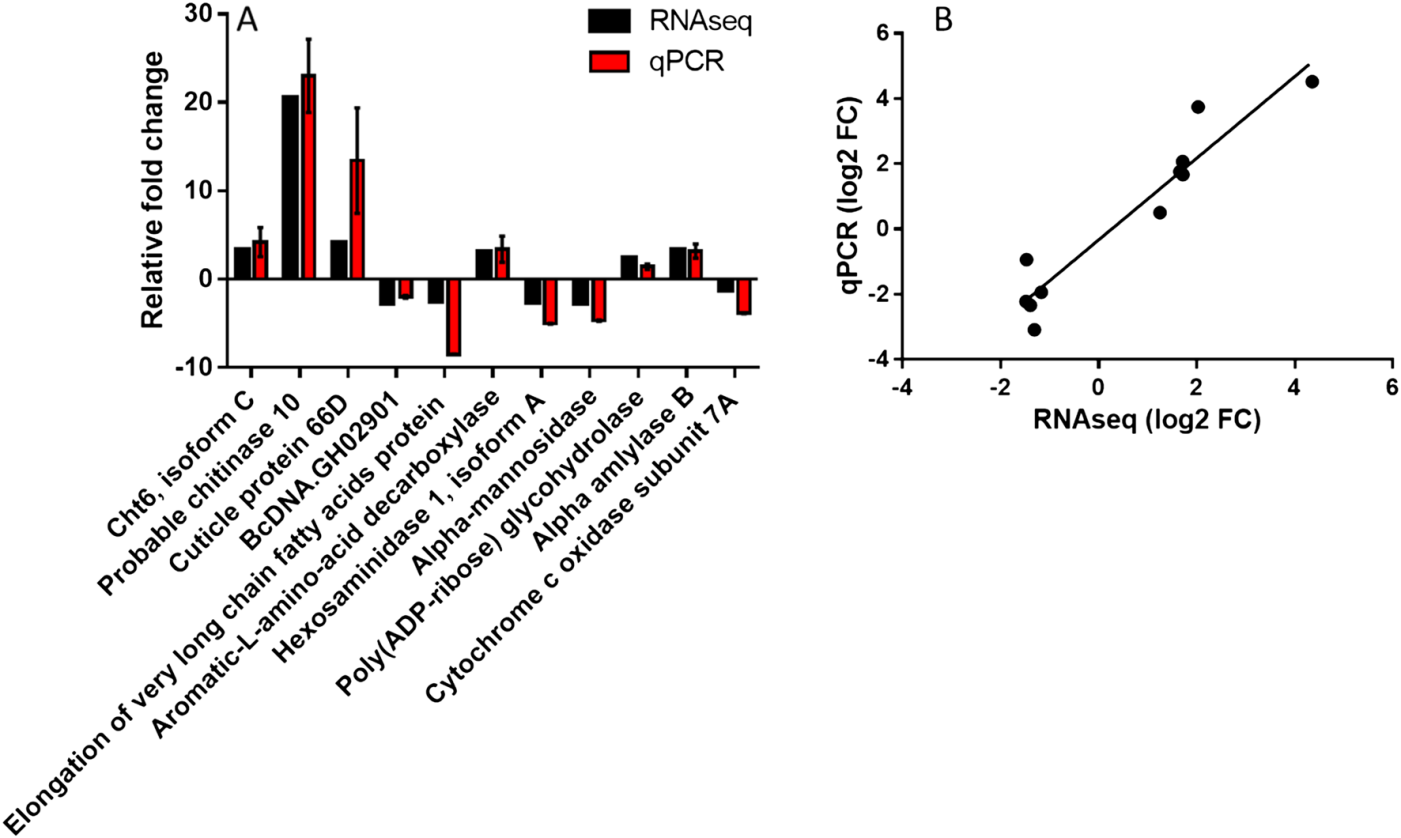
qPCR analysis confirming transcriptomic results. (**A**) The 11 genes selected for qPCR analysis showed overall consistency with RNA-seq despite some of the gene expression levels appeared stronger. (**B**) Correlation between RNA-seq and qPCR gene expression. EF1a is used as a housekeeping gene to calculate relative fold change.

To validate the RNA sequencing results, we used separate aliquots of the biological samples for sequencing along with two additional samples, and randomly selected 11 genes from either regime 2 or regimen 3 pairwise-GO enrichment analysis groups for quantitative real-time PCR confirmation. The genes chosen were significantly differentially expressed from both groups. The gene set is comprised of different functional groups and included 5 down-regulated genes and 6 upregulated genes. For both RNAseq and qPCR shown in Fig. 6A, genes cht 6 isoform C, probable chitinase 10, cuticle protein 66D, aromatic-L-amino-acid decarboxylase and alpha amylase B expressions are pairwise comparison of daily high soak (28.5 °C) relative to low soak (23.5 °C) in regime 2; whereas genes bcDNA.GH02901, elongation of very long chain fatty acids protein, hexosaminidase 1 isoform A, poly (ADP-ribose) glycohydrolase and cytochrome c oxidase subunit 7A are pairwise comparison of regimen 3: 23.5 °C relative to 24.5 °C. *EF1α* was used as an internal control for qPCR to normalize expression before pairwise comparison. While Fig. 6A showed that our qPCR results had relatively stronger expression levels in some genes (we added two more biological replicates in addition to those sent for RNAseq for qPCR analysis), overall the RNA-seq and qPCR results were consistent. Figure 6B shows the relationship between RNA-seq and qPCR gene expression (y = 1.245x − 0.31, R^2^ = 0.90). Primer sequences used for this validation are provided in Table S5).

## Discussion

The changing thermal regimes of freshwater ecosystems require that we better understand species responses to temperature at different time scales (e.g. hourly, daily, seasonally, annually). Recent efforts have made progress in our understanding of both short-term^36,37^ and long-term^38^ thermal challenge in *N. triangulifer.* Even so, the effect of ecologically relevant diel thermal variation with daily excursions into stressful temperatures on long-term survival and development remains poorly understood. It was critical for this study to establish an unambiguously stressful temperature in *N. triangulifer* (28 °C) such that we could explore the consequences of ecologically relevant transient exposures at both the levels of transcript expression and associated life history outcomes.

Previous studies in this species explored physiological processes associated with chronic thermal stress and indicated that lipid depletion, reduced trehalose synthase and increased histamine and heat shock protein (HSP) gene expression were associated with chronic thermal stress^38^. Here we opted for the RNA-seq approach so that we could obtain an unbiased view of transcript expression changes and assess even low-abundance transcripts^45–49^.

### Within regimen comparisons

Few (28) transcripts were differentially expressed in Regimen 1, where rearing temperatures fluctuated well within the typical temperatures experienced in the source population of this lab-reared clone. In contrast, mayflies reared under Regimen 2—with daily forays outside the thermal acclimation zone (diel fluctuations between 23.5 and 28.5 °C, daily mean 26 °C) resulted in a total of 614 significantly differentially expressed transcripts, with more transcripts being upregulated at the warmer temperature than down-regulated. Upregulated processes included the deactivation of rhodopsin mediated signaling and phototransduction. Rhodopsin has long been known as a light-sensitive receptor protein involved in visual phototransduction^50^. Interestingly, research have also showed that the rhodopsin signaling pathway is involved in light-independent roles, such as thermosensory signaling^51^. Moreover, a daily temperature oscillation (< 5 °C) within a physiological range synchronizes circadian rhythms in *D. melanogaster* and can also be independent from light-entrainable oscillators^52,53^. However, we suspect that in our case the enriched biological function is independent from thermal effects. Our sampling times and thermal fluctuation range (5 °C) were the same in pairwise comparison groups both within and outside the thermal acclimation zone. While transcript numbers were not sufficient enough for GO enrichment analyses for biological processes in the Regimen 1 comparison, some individual transcripts related to circadian clock and/or visual functions were also differentially expressed. This is not surprising because sampling time A is just after lights on (sunrise) and sampling time B is just before lights off (sunset), and are timed to important elements in a circadian rhythm or diel cycle. Therefore, this suggests that these GO enrichment analyses for biological processes observed for the variable 22 °C treatment are independent from thermal effects.

During the transient thermal stress in regimen 2, there was a reduction of transcripts related to *N*-glycan processing in the GO enrichment analyses for biological processes. *N*-glycan plays an extremely important role in proper protein folding, and the data suggests that this daily thermal push, while transient, is stressful enough to effect physiological processes at the molecular level in *N. triangulifer*. This is also consistent with our RNAseq data as well as previous findings, that chaperone protein HSP90 was upregulated under thermal stress to aid for proper protein folding^36^. Increased expression of transcripts associated with lipid catabolism and carbohydrate metabolism was observed during transient thermal stress. Reactome pathway analysis suggests that metabolism of lipids and lipoproteins is highly enriched, and that more than half of the transcripts involved in the pathway are enriched, supporting the GO results. These responses are consistent with previous metabolomic studies of chronic heat stress in *N. triangulifer*^36^ where depletion of certain lipids was observed. We did not find evidence of hypoxia signaling at chronic thermal limits (see^54–56^).

Transient thermal stress in regimen 2 also simultaneously stimulated chitin metabolic processes and chitin- based cuticle development. The exoskeleton of insects is an assembly of chitin and cuticle proteins^57^. Interestingly, nearly half of the top 20 most upregulated transcripts in this pairwise comparison group were related to chitin catabolism or cuticle development. It has been suggested that cuticle is sensitive to temperature changes^58–61^, and these changes in cuticular transcript expression are intriguing because our separate molting experiments suggest that the number of molts required to reach adulthood is “fixed”, with warmer temperatures simply accelerating the process. Under warmer conditions, larvae molt more frequently and at smaller body sizes than larvae reared under cooler conditions. Thus, molting is not determined by body size. It is interesting that even brief thermal ramping stimulates molting. Camp et al.^62^ observed that larvae were more likely to molt when put on the thermal ramp than when maintained at static temperatures.

Hormone signaling (i.e. juvenile hormone and ecdysteriods) and its involvement with cuticle proteins in regulating insect molting process has been well studied^63–65^. However, the linkage between thermal effects and molting frequency (perhaps through hormone signaling up-regulating cuticle protein genes or other unknown physiological mechanisms) has not been elucidated. The transcriptome analysis in our study may have revealed that thermal stress affects chitin catabolic process and molting cycle, still it remains unknown why mayflies tend to molt more frequently at warmer temperatures when the process of molting itself requires high energy cost. Regardless, the findings that total molts per life cycle is relatively fixed and time between molts decreases as temperature increases may help explain why mayflies result in smaller body size under warm temperatures (Fig. 1).

### Across regimen comparisons

A second goal of our study was to understand lingering effects of chronic but transient thermal stress. To achieve this goal, we compared transcript expression profiles between the warmer temperature of Regimen 1 (24.5 °C) and the cooler recovery temperature of Regimen 2 (23.5 °C) (regimen 3, e.g. the BC comparison in Fig. 3). We were surprised by how many genes were differentially expressed between these groups.

We found that the chronically stressed population had significant reductions in the expression of transcripts associated with ATP synthesis and the mitochondrial electron transport chain relative to the unstressed population. Both gluconeogenesis and glycolytic processes were simultaneously lower in the chronically stressed population. However, lipid biosynthesis was actually upregulated in the chronically stressed population, which is interesting because lipid catabolism was experienced in this population during the thermal challenge at 28.5 °C. It may be that the increased activity of lipid biosynthesis at the recovery temperature reflects a compensatory response to the heat stress. The finding of downregulation in energy metabolism was consistent with our previous study where we analyzed targeted metabolite end products in *N. triangulifer* exposed to chronic thermal stress statically^38^.

The other major difference between the two treatments was in the increased activity of neurotransmitter secretion, synaptic vesicle cycle and transport, and regulation of membrane potential. Many studies have provided evidence of hormonal and neurotransmitter change from the endocrinological aspect of insect stress response. Davenport and Evans^66^ linked the secretion of biogenic amines, which can function as neurohormones in response to stress. However, the stress response reaction depends on the speed of carbohydrate and lipid metabolic responses. The data from our pairwise comparison group in regimen 2 may suggest that while temperature rises slowly to a stressful level, the increase of energy-related metabolic processes to cope with that increasing thermal challenge may increase slowly as well. This helps explain the absences of neurotransmission related GO enrichment analyses for biological processesin regimen 2 (Figs. 3, 5). Interestingly, in our previous study where mayflies were subjected to chronic thermal stress, we found an increase in histamine and dopamine, both biogenic amines play the role as neurotransmitter^38^. Consistent with our current findings in this study, these data suggest that the thermal stress-induced neurological activities are also affected under chronically stressful conditions.

### Life history outcomes

We found that in regimen 1 (within the thermal acclimation zone), it did not matter if larvae experience constant vs. variable temperature. Survival, development time and adult sizes were not statistically different. However, when larvae are transiently but repeatedly pushed outside their thermal acclimation zone (regimen 2), both development time and fitness (inferred from adult body sizes) are negatively affected. A more detailed treatment life history outcomes across more thermal treatments is needed, however these results suggest that time spent at harmful temperatures is not offset by time spent at more ideal temperatures.

Together, our study shows that *N. triangulifer* larvae do not recover from daily forays into thermally challenging conditions. RNA-seq and GO enrichment analysis support our previous findings of energy source re-allocation under thermal stress. In addition, the study also emphasizes the role of molting in mediating thermal performance. Our study helps elucidate how a modest increase in daily thermal fluctuation affects transcript expression and its associated GO enrichment analyses for biological processes and ultimately life history outcomes in *N. triangulifer* and likely other aquatic insects*.*

## Supplementary information

Supplementary Table S1.

Supplementary Table S2.

Supplementary Table S3.

Supplementary Table S4.

Supplementary Table S5.

Supplementary Table S6.

Supplementary Table S7.
