# Supplementary material for: Transcriptomic and life history responses of the mayfly Neocloeon triangulifer to chronic diel thermal challenge
Source: Sci Rep. 2020 Nov 5;10:19119. doi: 10.1038/s41598-020-75064-y (PMC7644658; doi:10.1038/s41598-020-75064-y)

Supplemental table S7.

Transcriptomic and Life History Responses of the Mayfly *Neocloeon triangulifer* to chronic diel thermal challenge.

**Hsuan Chou^1^, Dereje D. Jima^2, 4^, David H. Funk^3^, John K. Jackson^3^, Bernard W. Sweeney^3^ and David B. Buchwalter^1^**^*^


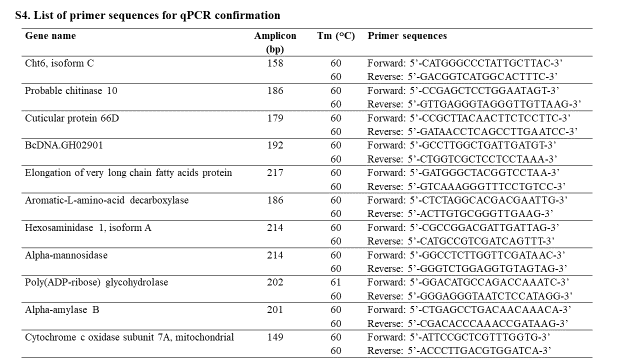

Supplement: Supplementary file 7 — Supplementary Table S7. [file 41598_2020_75064_MOESM7_ESM.docx]
